# Growth Mechanism for Low Temperature PVD Graphene Synthesis on Copper Using Amorphous Carbon

**DOI:** 10.1038/srep44112

**Published:** 2017-03-09

**Authors:** Udit Narula, Cher Ming Tan, Chao Sung Lai

**Affiliations:** 1 Center for Reliability Sciences & Technologies, Chang Gung University, 259, Wen-Hwa 1^st^ Road, Kwei-Shan, Taoyuan, 33302, ROC Taiwan; 2 Department of Electronic Engineering, Chang Gung University, 259, Wen-Hwa 1^st^ Road, Kwei-Shan, Taoyuan, 33302, ROC Taiwan; 3 Department of Mechanical Engineering, Ming Chi University of Technology, 84, Gungjuan Road, Taishan, New Taipei City, 24301, ROC Taiwan; 4 Department of Urology, Chang Gung Memorial Hospital, Kwei-Shan, Taoyuan, 33305, ROC Taiwan; 5 Institute of Radiation Research, College of Medicine, Chang Gung University, 259, Wen-Hwa 1^st^ Road, Kwei-Shan, Taoyuan, 33302, ROC Taiwan; 6 Department of Materials Engineering, Ming Chi University of Technology, Taishan, New Taipei City 24301, Taiwan; 7 Department of Nephrology, Chang Gung Memorial Hospital, Kwei-Shan, Taoyuan 33305, Taiwan

## Abstract

Growth mechanism for synthesizing PVD based Graphene using Amorphous Carbon, catalyzed by Copper is investigated in this work. Different experiments with respect to Amorphous Carbon film thickness, annealing time and temperature are performed for the investigation. Copper film stress and its effect on hydrogen diffusion through the film grain boundaries are found to be the key factors for the growth mechanism, and supported by our Finite Element Modeling. Low temperature growth of Graphene is achieved and the proposed growth mechanism is found to remain valid at low temperatures.

Graphene has been a material of great interest since its conception due to its extraordinary properties such as its single atomic thickness^1^, high current density tolerance^2^ of about 10^8^ A/cm^2^, extremely high mobility^3^,^4^ of about 15,000 cm^2^/Vs, high thermal conductivity^5^ in the order of 5 × 10^3^ W/mK, high optical transmittance of about 97% within the visible light wavelength-range of 400 to 700 nm^6^, high mechanical strength with breaking strength of 42 Nm^−1^ and second and third-order elastic stiffness of 340 Nm^−1^ and −690 Nm^−1^ respectively^7^.

Over a decade, Graphene synthesis methods have witnessed many pathways in order to produce high quality Graphene with a high degree of coverage area. Currently, the Graphene synthesis method used is Chemical vapor deposition (CVD) technique^8^,^9^,^10^,^11^ which produces uniform, large area, relatively low defect Graphene films and is known to be the cheapest for mass production and can meet the semiconductor industry requirement^11^.

Growth of Graphene on Copper using CVD has been demonstrated by Li *et al*.^11^,^12^ and others^13^,^14^. However, this method uses expensive carbon source which is also wasted considerably during chamber cleaning. This method also have no control over number of layers of synthesized Graphene when it comes to few‐layered and multi‐layered graphene growth.

On the other hand, a simpler and low cost technique to obtain non-transferable Graphene using amorphous carbon as solid source and Nickel or Cobalt thin film as catalyst annealed at elevated temperatures has been reported^15^,^16^,^17^. This technique can be instrumental in controlling the number of Graphene layers, eliminates the need for Graphene transfer and is economical as it uses amorphous carbon as the carbon source. Non-transferable Graphene synthesis is important especially for ULSI Interconnects which are used in BEOL (back-end-of-line) process and transfer of Graphene is quite infeasible. Moreover, the transfer of Graphene introduces many structural inhomogeneities which causes degradation of properties of graphene such as the mobility, electrical and thermal conductivity etc^18^,^19^,^20^. However, such Graphene synthesis method was reported to be not possible with Copper due to the very low solubility of Carbon in Copper^16^,^17^.

As the applications of Copper material is vast and important, such as printed circuit boards, transmission wires and cables, its application in ULSI interconnects, etc, the growth of Graphene on Copper (Cu) can overcome some limitations of Cu materials such as its ease of corrosion, ease of oxidation, limited Electromigration performance as interconnect, and push the electrical and thermal conductivity limits of Cu to higher values.

In view of the limitation of the reported methods, the use of amorphous carbon as solid source for Graphene synthesis on Cu was re-explored by Ji *et al*.^21^. However, their method involved Graphene deposition on a copper foil which is several microns thick and is not suitable for semiconductor industry as the Graphene so obtained needs to be transferred on to the required substrate which might introduce defects. Also, the Graphene is in direct contact with amorphous carbon in the method used by Ji *et al*.^21^, as the amorphous carbon layer atop Cu crystallizes to Graphene and this might result in crystal contaminations.

Recently, we successfully demonstrated the feasibility of crystallization of amorphous Carbon under sputtered Copper thin film which acts as a catalyst in order to obtain Graphene at the top surface of Cu experimentally^22^. However, its growth mechanism is yet to be explored.

In this work, various experiments are designed and performed in order to explore the growth mechanism of our Physical Vapor Deposition (PVD) Graphene growth method using amorphous carbon as the carbon-source. The proposed growth mechanism can also explain the claim of inability of Graphene growth on Copper thin film using this method as reported by others^16^,^17^. An attempt to achieve similar synthesis at comparatively lower temperature is presented and the growth mechanism is discussed for the same.

## Methods

### Sample Preparation

**Figure 1:**
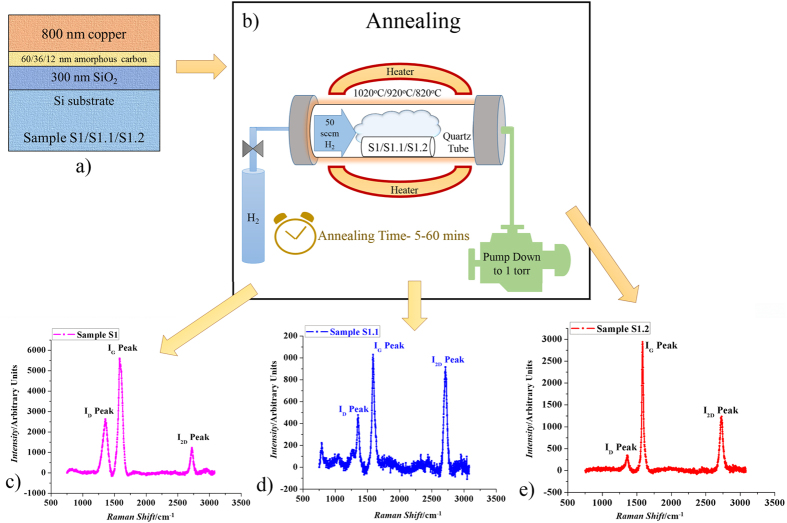
(**a**) Structure design for samples S1, S1.1 and S1.2 having 60 nm, 36 nm and 12 nm a-C layer sandwiched between 800 nm Cu and Si/SiO_2_(300 nm) substrate, (**b**) Schematic for annealing process, (**c**) Raman Spectrum of annealed sample S1, (**d**) Raman Spectrum of annealed sample S1.1, (**e**) Raman Spectrum of annealed sample S1.2^22^.

The experiments in this work involve deposition of amorphous Carbon (a-C) thin film of different thicknesses followed by Copper (Cu) (99.99%) film of 800 nm thickness on Si/SiO_2_ (300 nm) substrates using Physical Vapor Deposition (PVD) method. The samples are labelled as S1, S1.1 and S1.2 respectively as shown in Fig. 1a)^22^. (For sample preparation schematic and surface condition, please refer to Supplementary Figures S1 and S2 respectively in the Supplementary Information).

### Annealing

After samples S1, S1.1 and S1.2 are produced, they are annealed in an H_2_ environment with a flow rate of 50 sccm at a low pressure of 1 torr and different annealing temperatures and annealing times as shown in the annealing process schematic in Fig. 1b).

The graphene synthesis method used has been described in detail by Narula *et al*.^22^.

### Characterization

Raman scattering spectra are collected using PTT RAMaker Micro Raman/PL/TR-PL Spectrometer with laser excitation wavelength of 473 nm and laser spot-size of about 0.5 μm.

## Results

In order to understand the underlying mechanism of this graphene growth, experiments and characterization are performed to examine the effect of thickness of a-C, annealing time and annealing temperature for Graphene crystallization on Cu. In order to avoid any changes or modifications in the properties of synthesized Graphene, the experimental analysis is performed completely on the growth substrate. Therefore, all the reported data have been obtained with graphene still being on the Copper thin film.

### Effect of a-C layer thickness on Graphene growth

Figure 1c,d and e show the Raman spectra of the annealed samples S1, S1.1 and S1.2 (each having different a-C thicknesses) respectively, and Table 1 summarizes their Raman characteristics^22^.

It is well known that the I_G_ peak is associated with the doubly degenerate phonon mode at the Brillouin zone center which comes from a normal first order Raman scattering process in graphene and originates from the interplanar sp^2^-bonded C-C stretching vibrations^23^. The I_2D_ and I_D_ peaks originate from a second-order process, involving two iTO phonons near the K point for the 2D band and one iTO phonon and one defect in the case of the D band which can be often used to evaluate the number of graphene layers and the grain size of graphene respectively^23^.

From these information, we can see that in Fig. 1d, the I_2D_/I_G_ peak ratio of sample S1.1 is much higher (close to 1) than that of sample S1 (Fig. 1c), and its I_D_/I_G_ peak ratio is nearly the same as S1. As for S1.2 (Fig. 1e), its I_2D_/I_G_ peak ratio is also higher (but less than 1) than sample S1, and its I_D_/I_G_ peak ratio is much less as compared to S1. I_2D_/I_G_ peak ratio equal or greater than 1 indicates the presence of few layers (i.e. layer number around 3–9) of Graphene while the peak ratio less than 1 indicates multi-layer Graphene (i.e. layer number more than 10)^24^. As all the three samples have I_2D_/I_G_ peak ratio less than 1, their graphene have multi-layer, with sample S1 having the largest number of layer, and sample S1.1 having the least number of layer, despite that the a-C thickness of S1.1 is not the least. This will be explained later from our proposed growth mechanism.

### Effect of annealing time on Graphene growth

**Figure 2:**
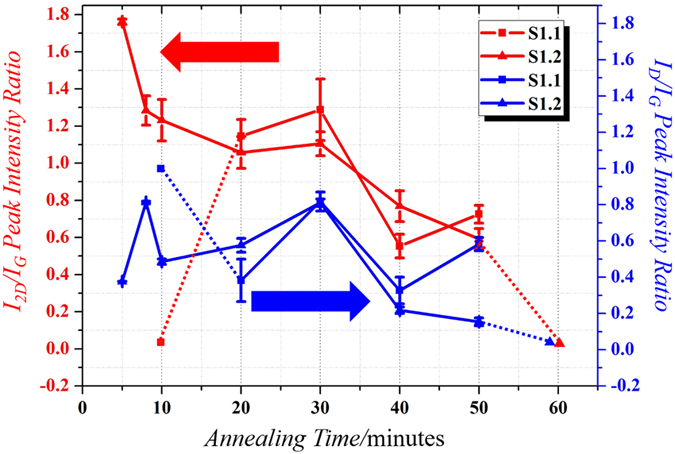
I_2D_/I_G_ and I_D_/I_G_ vs anneal time measured by Raman Spectroscopy. Red lines are for the I_2D_/I_G_ peak ratio with the scale on the left y-axis, and blue lines are for the I_D_/I_G_ peak ratio with the scale on the right y-axis. Dotted lines indicates the uncertainty of the trend as mentioned in the text.

**Figure 3:**
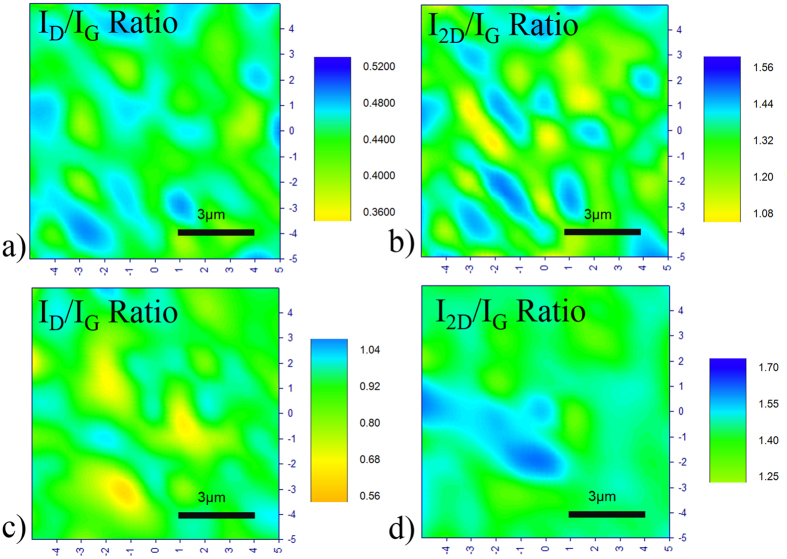
Raman Mapping of 10 μm × 10 μm area recorded in steps of 1 μm in all directions for sample S1.1 annealed at 1020 °C for (**a,b**) 20 minutes and (**c,d**) 30 minutes.

**Figure 4:**
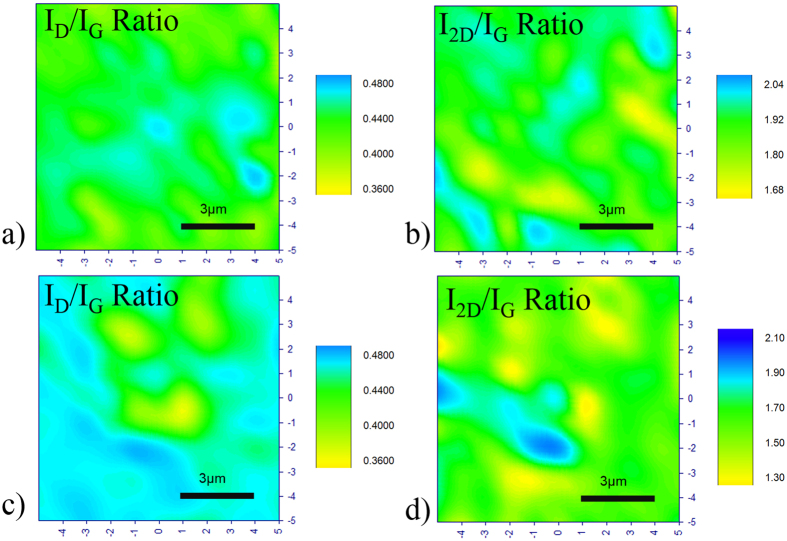
Raman Mapping of 10 μm × 10 μm area recorded in steps of 1 μm in all directions for sample S1.2 annealed at 1020 °C for (**a,b**) 5 minutes and (**c,d**) 8 minutes.

Samples S1.1 and S1.2 are subjected to annealing with different annealing times, and Raman signatures with higher 2D peak are indeed observed. Figure 2 shows the plot of I_2D_/I_G_ and I_D_/I_G_ vs annealing time. Figures 3 and 4 shows the Raman Maps for samples S1.1 and S1.2 respectively for different annealing times. The means plotted with error bars in Fig. 2 are consistent with the Raman Mappings in Figs 3 and 4.

As I_2D_/I_G_ ratio greater than 1 represents the presence of few layers of graphene, a sharp increase in the ratio is expected from zero (where no graphene is present) to a value much greater than 1, followed by a decrease in the ratio towards less than 1 as the number of graphene layer is increasing. Likewise, when the graphene layer is being etched away, a sharp change in the ratio from some values less than 1 to a value much greater than 1 and then down to zero should be expected. However, due to the discrete annealing time used in our experiments, such sharp change in the ratio is difficult to observe, and only dotted lines are shown in Fig. 2 for the period where these sharp changes are expected.

For sample S1.1, no Graphene is present for 10 minutes of annealing time and its surface layer is highly defective as indicated by its I_D_/I_G_ ratio. Graphene formation can be found with 20 minutes of annealing time while the I_D_/I_G_ ratio drops to a lower value. The largest I_2D_/I_G_ ratio is observed with 30 minutes of annealing time which is a signature of few layers Graphene (since I_2D_/I_G_ ratio is higher than 1) and an increase in I_D_/I_G_ ratio can also be observed. This is followed by a drop in I_2D_/I_G_ and I_D_/I_G_ ratio with 40 minutes of annealing time and then a small rise in the I_2D_/I_G_ for 50 minutes of annealing time while the I_D_/I_G_ ratio also increases at this point.

On the other hand, the thin a-C layer in sample S1.2 undergoes early crystallization within 5 mins of annealing to produce few layers of Graphene which continues to form multi layered Graphene with a small increase in I_2D_/I_G_ ratio during 20 to 30 minutes of annealing time. Beyond 30 minutes of annealing time, its I_2D_/I_G_ and I_D_/I_G_ ratios decrease continuously, in contrast to sample S1.1, and approaches zero for 60 minute of annealing time. The defects in sample S1.2 is much less than that in sample S1.1 for annealing time more than 40 minutes.

In the light of the above findings, we can understand the reason of the conclusion made by the reported works on the infeasibility of crystallization using Cu as catalyst because they were either having annealing time less than 10 minutes and/or the thickness of a-C film is too thick where the formation of graphene formation will begin only at longer annealing time^17^.

### Effect of annealing temperature on Graphene growth

**Figure 5:**
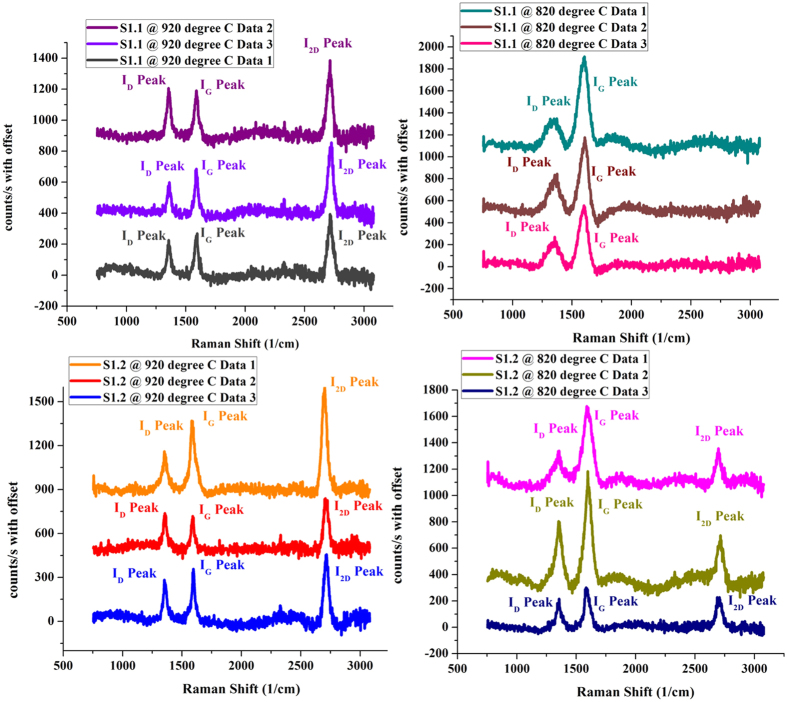
Plots of Raman spectrum for sample S1.1 and S1.2 annealed at 920 °C and 820 °C for 50 mins.

Samples S1.1 and S1.2 are annealed comparatively at lower temperature of 920 °C and 820 °C for 50 mins. Figure 5 shows plots of Raman spectrum for sample S1.1 and S1.2 annealed at 920 °C and 820 °C for 50 mins. It is evident from Fig. 5 that Graphene with higher 2D peak can be grown in low temperature regimes. However, for sample S1.1, Graphene is no longer synthesized at 820 °C, in contrast to for sample S1.2. No graphene is observed in both samples with further reduction in annealing temperature to 800 °C.

The distinct difference in the behavior of the graphene formation with annealing time and temperature for S1.1 and S1.2 is interesting since the difference in the two samples is only the thickness of the a-C layer. This kind of distinct behavior is believed to be due to the thermo-mechanical stress driven crystallization of a-C into Graphene, and we would like to verify it using finite element analysis.

### Finite Element Analysis

Finite Element Analysis (FEA) is performed using ANSYS Workbench R16.2 platform. As thermo-mechanical stress^25^ is given as:


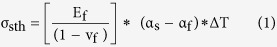


where *E*_*f*_ and *v*_*f*_ are the Young’s modulus and Poisson’s ratio respectively, *α* is the coefficient of thermal expansion, and Δ*T* is the range of temperature excursion; the subscripts ‘*s’* and *‘f’* refer to substrate and film, respectively, we can see that the stress is independent of the thickness of the a-C layer, and thus the thermo-mechanical stresses in each sample are the same. On the other hand, the thermo-mechanical stress causes the sample to warp, and the resultant Maximum Principal Stress is given by the Stoney’s equation^26^,^27^ given as:


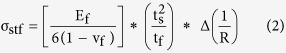


where Δ*(1/R)* = (*1/R−1/R*_*0*_), and *R*_0_ and *R* are the radius of curvature of the samples before and after the thermo-mechanical stresses are developed respectively. ‘*t’* is for thickness of the layer in the sample with subscripts *‘s’* and *‘f’* refer to substrate and film, respectively. Here Maximum Principal Stress is considered in the Stoney’s equation for a-C as a-C is known to be a brittle material^28^.

As Δ(1/R) is due to the warpage of the sample caused by thermo-mechanical stress which is independent of layer thickness in the sample, the value of Δ(1/R) can be assumed to be a constant for the three samples.

**Figure 6:**
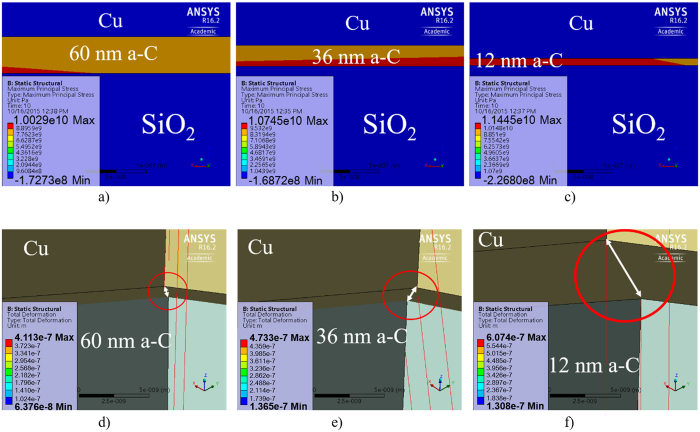
ANSYS simulation results of (**a–c**)^29^ Maximum Principal Stress distributions in the three samples at 1020 °C, zoning into the maximum stress areas; the area of maximum stress distribution is the largest in S1.2 and the least in S1, and the stresses are compressive, (**d–f**) Von-Mises stress in Cu (tensile) at the corner of each sample, showing the delamination at the a-C/substrate and Cu/a-C interfaces in these samples; maximum delamination can be observed in sample S1.2.

According to Equation (2), the Stoney’s equation indicates that thin film stress is inversely proportional to film thickness, with samples S1, S1.1 and S1.2 having a-C thicknesses of 60 nm, 36 nm and 12 nm respectively, the total stress in sample S1.2 should be the highest and that in sample S1 should be the lowest, as indeed shown in Fig. 6a–c 29 as computed by ANSYS. The thermo-mechanical properties of the materials used in the simulation are shown in Supplementary Table S1. Figure 6a–c 29 shows the comparison of the Maximum Principal Stress distributions in the three samples at 1020 °C, and the maximum stress values in a-C layers are decreasing from sample S1.2 to S1.1 and then to S1 as expected from the Stoney’s equation.

Figure 6d–f shows the delamination of Cu and a-C films from their respective substrates at the corners where stresses are the highest within each sample. Here we consider Equivalent Von-Mises Stress analysis for Cu because Cu is ductile in nature^30^. Again, we can see that the delamination is most severe for S1.2 due to the presence of highest stress in Cu and a-C layer. The von-Mises stresses in Cu film are tensile in nature and their values are believed to be high enough that can either cause cracking in Cu film and/or enhancement of H_2_ diffusion in Cu film through its grain boundaries^31^,^32^,^33^.

**Figure 7:**
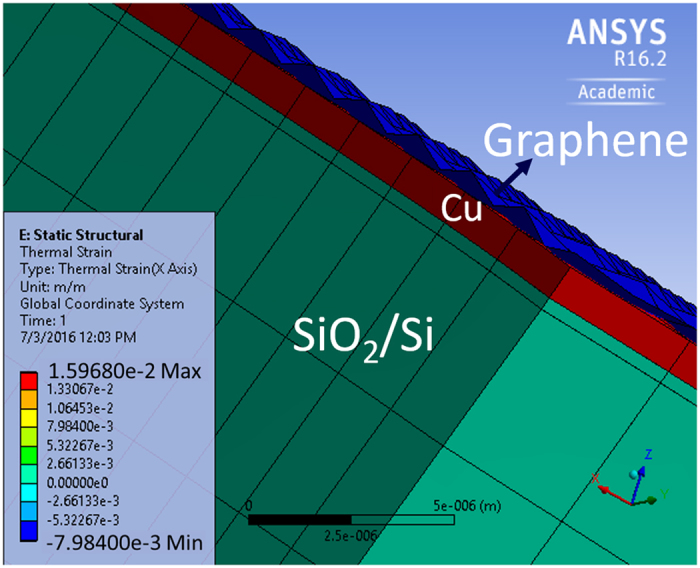
ANSYS simulation of thermal strain in Graphene on Cu at 1020 °C; Cu film possesses tensile strain whereas Graphene possesses compressive strain.

Furthermore, thermal strain analysis has also been performed on synthesized Graphene on Cu using ANSYS and is depicted in Fig. 7. The simulation results suggest that the Cu thin film possesses tensile strain (positive strain value) while the Graphene layer possesses compressive strain (negative strain value).

Based on our FEA simulation results, we revisit and explain our experimental results in the following discussion.

## Discussions

**Figure 8:**
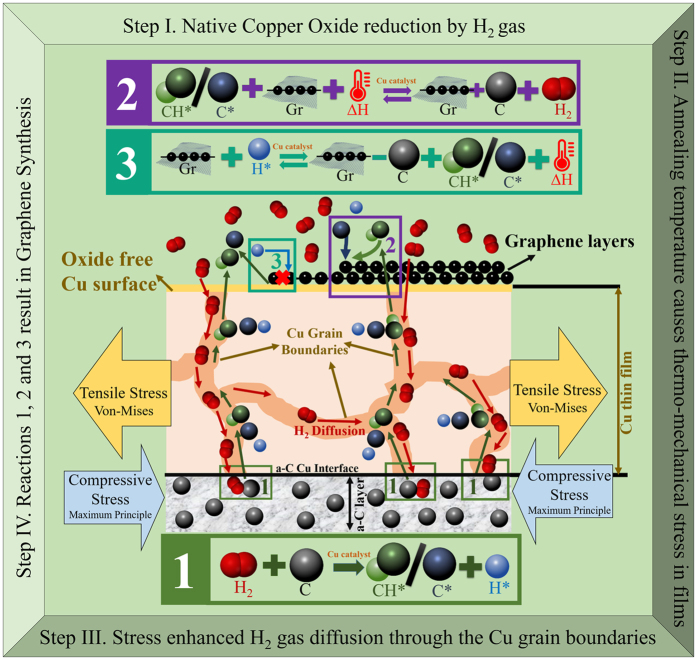
Governing reactions^29^ for Graphene growth forming the basis for proposed mechanism of Graphene synthesis on Copper film; Here C represents Carbon atom, Gr represents Graphene, CH*/C* represent Hydrogen bonded-Carbon radical/Carbon radical, H* represent Hydrogen radical and ∆H represents heat.

Sample S1 has the most number of Graphene layers as observed and shown in Fig. 1c. In the same token, S1.2 should have the least number of Graphene layers as its a-C layer thickness is minimum, but Fig. 1e shows otherwise. This abnormality can be better understood by analyzing and comparing the trends of I_2D_/I_G_ and I_D_/I_G_ ratio, (as shown in Fig. 2) with the aid of the following governing chemical reactions for graphene growth as given by Vlassiouk *et al*.^34^. These reactions are shown in Fig. 8 and are modified for our experiments^29^ since methane is not used. Formation of hydrocarbons responsible for Graphene formation by the reaction of a-C and H_2_ at elevated temperatures as shown in Reaction (1) has been confirmed by Ji *et al*.^21^ as well.

For annealing time below 10 minutes, the Graphene obtained for sample S1.1 is totally defective as indicated by the nearly zero I_2D_/I_G_ peak and very high I_D_/I_G_ in Fig. 2. This is believed to be due to the lower tensile stress in Cu film, and hence longer annealing time is required for hydrogen to reach the underneath a-C for Graphene crystallization. Consequently, defective Graphene is observed below 10 minutes.

As annealing time increases to 20 minutes, the crystallization rate increases with time. The increase in crystallization rate is according to Reaction (2)^29^ in Fig. 8, which is an endothermic reaction^32^. As H_2_ gas has a dual role of carbon activator and Graphene etchant, etching of graphene by the H_2_ gas occur concurrently, and the number of Graphene layers is reduced. With the availability of the Hydrogen radicals, the exothermic etching reaction (Reaction (3)^29^ in Fig. 8) is shifting in the forward direction^35^. The combined effect of crystallization and etching of Graphene results in formation of few layers of Graphene, which can be observed by an increased value of I_2D_/I_G_ ratio beginning from the 20 minutes of annealing. Due to discretization of annealing times used in our experiment, the exact extent of crystallization and etching of Graphene during 10 to 20 minutes of annealing times are unknown. For 20 minutes of annealing, the quality of Graphene film is improved as compared to that with 10 minutes of annealing and is less defective as reflected by a decreased value of I_D_/I_G_ value. The production of carbon radicals is also accompanied by production of hydrogen radicals as per the Reaction (1)^29^ in Fig. 8.

As the annealing time increases to 30 minutes, the amount of hydrogen radicals increases to sufficient quantity to trigger Reaction (3). Also the heat of absorption due to Reactions (1) and (2) reduces the temperature of the Cu film surface. As a result, the crystallization rate is dominated by the etching rate of Graphene, and thus Graphene is continuously etched by H_2_, primarily at defective sites because these sites facilitates preferential etching of Graphene^36^. This results in an even lesser number of Graphene layers than the previous shorter annealing times with increased defective sites. Hence, a continuous increase in the I_2D_/I_G_ and I_D_/I_G_ ratios up to 30 minute of annealing time in Fig. 2 is observed.

When the annealing time goes beyond 30 minutes, the remaining graphene layer is so thin and the heat generated from Reaction (3) plus the increasing amount of carbon radicals as a by-product of Reaction (3) enhance the crystallization Reaction (2) in the forward direction. This facilitates further crystallization, and more number of Graphene layers is formed as indicated by a decrease in I_2D_/I_G_ ratio, by the end of 40 minutes of annealing in Fig. 2. Also a decrease in I_D_/I_G_ is observed because the newly formed layers fill the defective sites created due to etching in the earlier annealing process.

For annealing time beyond 40 minutes, the scenario is similar to what happened during 20 to 30 minutes of annealing time where the hydrogen radicals are also produced with carbon radicals. This increased number of hydrogen radicals and reduction of the Cu film surface temperature because of heat of absorption due to Reactions (1) and (2) triggers Reaction (3) and shifts it in the forward direction, resulting in reduction in the number of Graphene layers. Thus only few layers of Graphene are formed by the end of 50 minutes of annealing. Also the defects increase due to loss of carbon atoms from the Graphene crystal forming defective sites. This is indicated by an increase in I_2D_/I_G_ and I_D_/I_G_ ratios in Fig. 2.

Similar behavior depicted in Fig. 1e for sample S1.2 may be explained with the help of Fig. 2. One may attribute the phenomena to the higher tensile stress in Cu film that enhance hydrogen diffusion through Cu film and graphene synthesis with fairly low I_D_/I_G_ ratio as can be observed even for annealing time as low as 5 minutes in Fig. 2. Due to higher tensile stress in S1.2, the carbon radical supply onto the Cu surface is high because of the enhanced hydrogen diffusion through the Cu film, renders Reaction (2) favorable and thus graphene growth continues. An increase in number of graphene layers is observed for annealing times between 8 to 20 minutes as indicated by the decrease in I_2D_/I_G_ ratio in Fig. 2. The I_D_/I_G_ ratio also decreases because the newly formed layers fill up the defective sites, thus reducing the effective defects in the Graphene crystal so formed. The high rate of Reaction (1) produces more H*, and the heat of absorption due to Reactions (1) and (2) reduces the temperature of the Cu film surface. These two factors enhance Reaction (3) in its forward direction and reduces the layer of graphene synthesized when the annealing time is prolonged to 30 minutes. Thus, the number of graphene layer is no longer increasing continuously with the annealing time, and a slow reduction in the number of layer takes place as Reaction (3) starts dominating slowly, and this is indicated by a small increase in I_2D_/I_G_ while the I_D_/I_G_ ratio also increases due to etching of graphene as explained before. Thereafter, the growth Reaction (2) becomes active swiftly for the annealing time beyond 30 minutes, and I_2D_/I_G_ and I_D_/I_G_ ratio keep on decreasing. This is because the Reaction (3) in the previous step produces carbon radicals as a by-product and Graphene growth Reaction (2) will be shifted to its forward reaction, giving hydrogen radicals lesser chance to etch away the graphene. When the annealing time is long enough (40 minutes and beyond), unlike the situation in sample S1.1, a different mechanism kick in as can be seen from the different rate of change of the I_2D_/I_G_ ratio as seen in Fig. 2. This is believed to be due to two possible mechanisms.

**Figure 9:**
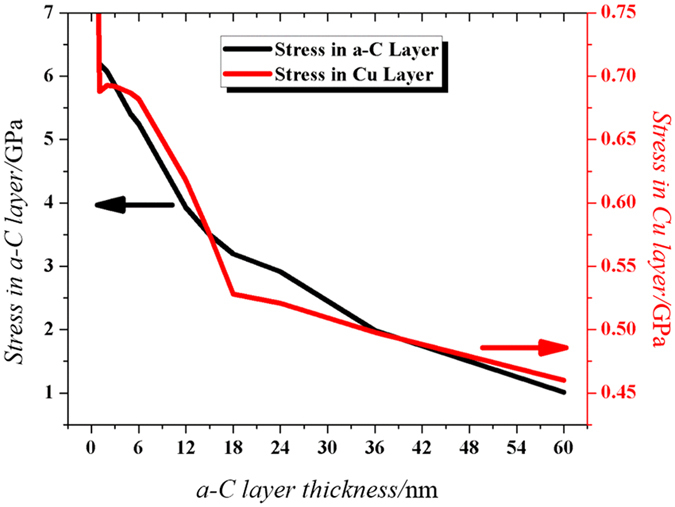
Evolution of stress with decrease in a-C layer thickness.

**Figure 10:**
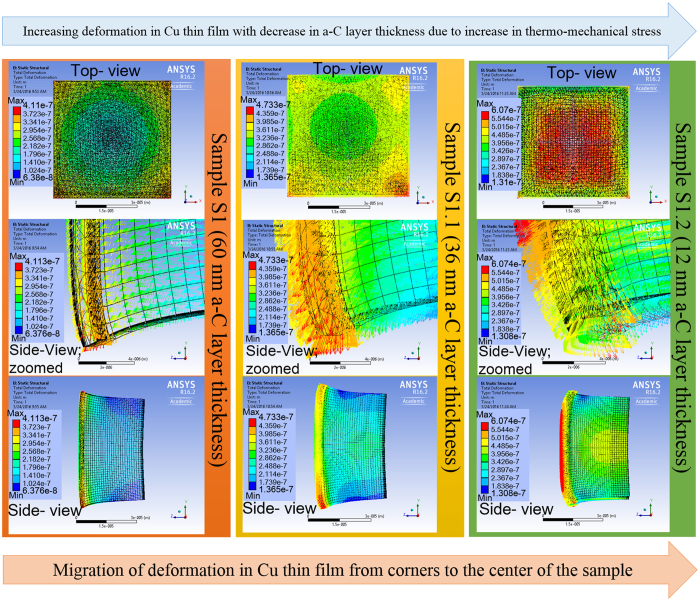
ANSYS simulation results for samples S1, S1.1 and S1.2 showing total deformation in the samples which migrates from the corners to the center with the decrease in a-C layer thickness.

One mechanism is stress induced migration^37^ or stress induced voiding^38^,^39^ in Cu that enhance the movement of vacancies in Cu film. These voids move outwards toward the edge of the Cu film and during this movement, they combine and form bigger voids, enhancing the diffusion of hydrogen and carbon radical supply. The rationale behind this postulation is derived from the computed tensile stresses in Cu film on SiO_2_ substrate and on a-C film with SiO_2_ substrate. Our finite element calculations using Equations (1) and (2) show that the tensile stress in Cu film for the former is 1190 MPa, and that for the latter ranges from 460 MPa to 690 MPa depending upon the a-C layer thickness as depicted in Fig. 9. With a-C film thickness of 12 nm at the beginning for S1.2 sample, and after 40 minutes of annealing, the a-C film thickness has become very small, and hence the tensile stress in Cu film could be sufficiently high for stress induced voiding to occur, especially under elevated temperature during annealing. This rationale is further strengthened by the ANSYS simulation for the deformation in the samples shown in Fig. 10. It shows that the maximum deformation which is a direct measure of area under high stress, is distributed at the corners for sample S1 and S1.1 while the same is densely distributed at the center for sample S1.2. This is because initially the corners are comparatively free to deform so the high values of deformation concentrate there. With the decrease in a-C layer, the stress at a-C/Cu interface starts to increase slowly causing delamination at the corners as shown in Fig. 6d–f. Hence, the corners become stress free and the deformation to migrate from the corners to the center area resulting in higher stress distribution at the center. Therefore, the carbon radicals move out slowly from the corners in S1.1 and with the decrease in a-C layer thickness, the movement of the radicals become higher from the center. In other word, the movement of carbon is large at the center of S1.2 in the beginning, and the distribution area is wide which keeps on widening.

Another mechanism is the sharp increase in the a-C film compressive stress as computed using finite element as shown in Fig. 6. When the annealing time is longer than 40 minutes, the remaining thickness of a-C layer could be below 4 nm for S1.2 since its initial thickness is only 12 nm, and the stress in a-C layer becomes very high as depicted in Fig. 9. This large compressive stress in a-C layer will “push” the carbon atoms in perpendicular directions in order to release the stress, enhancing both the hydrogen and carbon diffusion^29^.

As a result of the above two mechanisms, the rate of crystallization of Graphene is much faster than the rate of etching, and the number of layer of the synthesized Graphene increases at a higher rate in S1.2, resulting in multi-layered graphene formation. With such a higher rate of diffusion of carbon radicals and formation of graphene, and the thin layer of a-C in S1.2 at the start, when annealing time go up to 60 minutes and beyond, the remaining a-C is quickly depleting. As a result, the Reaction (2) shifts in the backward direction and Graphene etching reaction becomes dominant and hence all the grown Graphene is etched away by H_2_ gas. The points at which the growth of Graphene layers stops and etching reaction takes over is unknown due to discretization of annealing times in our experiments.

On the other hand, the above-mentioned two mechanisms are unlikely to happen in S1.1. First of all, its a-C thickness is 36 nm to start with, and after 40 minutes of annealing, its thickness is still well above 4 nm, and thus the increase in the compressive stress of a-C is slow as seen in Fig. 9. Also, with thick a-C film still underneath, the tensile stress in Cu film is unlikely to increase significantly to trigger the stress induced voiding.

**Figure 11:**
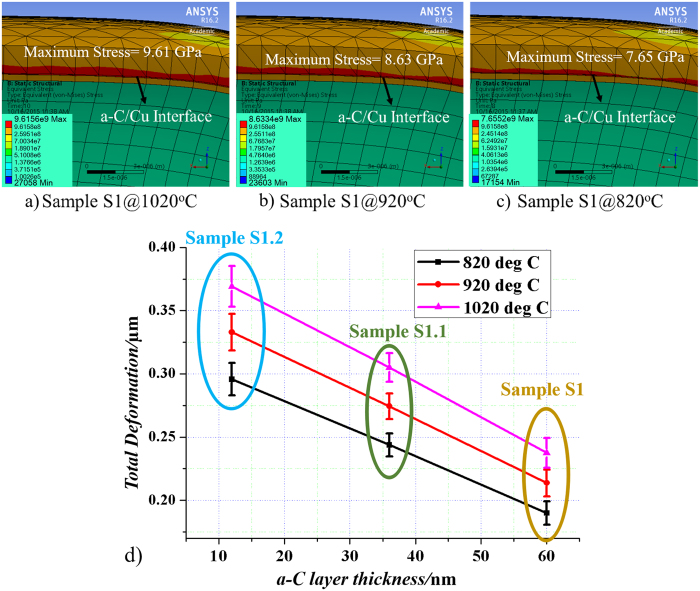
ANSYS simulation results (**a–c**) maximum von-Mises stress at a-C/Cu interface for sample S1 with respect to different annealing temperatures, (**d**) plot of total deformation in samples S1, S1.1 and S1.2 vs different annealing temperatures.

When the annealing temperature is lowered, the value of the von-Mises stress in Cu film and the area of maximum stress are reduced. Figure 11a–c shows the ANSYS simulation results of the von-Mises at different annealing temperatures for S1, and one can see a reduction of the maximum stress area, and the values of the maximum stress decreases from 9.61 GPa to 7.65 GPa as the temperature decreases from 1020 °C to 820 °C. Thus, the enhanced hydrogen diffusion through Cu film decreases significantly which slow down the reaction between H_2_ and a-C. Consequently, carbon radicals’ supply is reduced but Graphene etching by H_2_ gas still persists. Thus, only formation of nearly single layer or very few layers Graphene are possible.

When the temperature is further lowered, the diffusion of H_2_ through the Cu film will be so slow that Reaction (1)^29^ (mentioned in Fig. 8) will proceed very slowly, resulting in much slower Reaction (2), and the Graphene so formed will be etched away immediately by the H_2_ gas on top (as H_2_ gas diffusion through Cu film is also slow down, and thus its surface concentration will be high for etching). Thus no formation of graphene layers is observed below a certain temperature, where we term it as threshold temperature. This threshold temperature is lower for S1.2 since it has thinner a-C film and thus the stress in Cu film is higher than S1.1 for a given temperature as shown earlier, and this is consistent with our experimental results. ANSYS simulation for total deformation as shown in Fig. 11d validates the fact the stress in Cu for sample S1.2 is the highest followed by sample S1.1 and S1 even at lower temperatures.

Moreover, the growth mechanism discussed for annealing temperature value of 1020 °C stands true for lower temperature regimes as well because the trends in stress variation are similar to the high temperature regimes. (For more details, please refer to Supplementary Figures S3,S4 and S5 in Supplementary Information).

**Figure 12:**
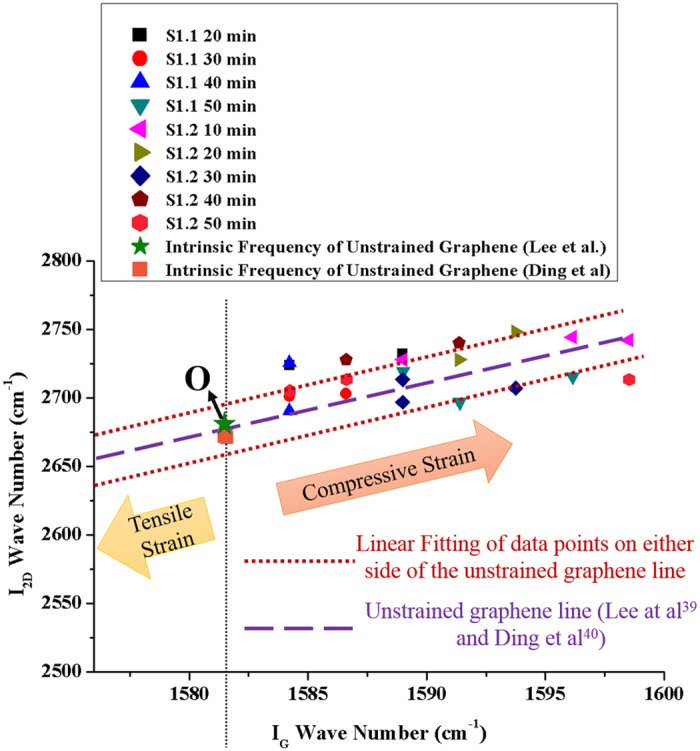
Relation between thermo-mechanical strain and Raman frequencies of G (ω_G_) and 2D (ω_2D_) modes; The data points for samples S1.1 and S1.2 (represented by red-dotted linearly fitted line) are lying parallel to the unstrained graphene line^39^,^40^ (purple dashed line) and indicate compressive strain.

The thermal strain results presented in Fig. 7 are verified using Raman characteristics as shown in Fig. 12. Figure 12 represents the plot of Raman frequencies of G (ω_G_) vs 2D (ω_2D_) modes. Dashed line in purple color with a slope (Δω_2D_/Δω_G_) of 2.2 represents Unstrained Graphene^40^ and point ‘O’ represents intrinsic frequencies of the two modes which are not affected by strain or excess charges^40^,^41^. The chances of doping in our samples are minimum as the experiments are conducted in vacuum. The Raman frequencies of G and 2D modes for samples S1.1 and S1.2 (represented by red-dotted linearly fitted lines) are nearly parallel to the Unstrained-Graphene line and the belong to Compressive Strain region^40^.

A worth-noting point observed in our samples is that the I_D_ peak intensity, which is also connected with defects, is on a higher side but these defects are advantageous with respect to contact resistance on metals^42^,^43^, and thus will be beneficial for ULSI Interconnects’ applications.

### Proposed Mechanism

From the above investigations, we can see that graphene synthesis on Cu is having a different mechanism as compared to that in the conventional CVD and gaseous carbon-source based graphene synthesis. Based on the results obtained in this work, we propose the plausible mechanism for the Graphene synthesis using amorphous carbon as the solid carbon source in presence of copper catalyst as depicted in Fig. 8 which consists of four steps.

*Step I-* The process begins with native copper oxide reduction by H_2_ gas at high temperatures ranging from 820–1020 °C, releasing water molecules in the gas phase as by-product and stress induced grain growth^44^,^45^.

*Step II-* The high annealing temperature causes thermo-mechanical stress to develop in the films of the sample, and its subsequent warping, rendering high tensile stress in the Cu film.

*Step III*- The high tensile stress due to thermo-mechanical stress in Cu film then enhances hydrogen gas diffusion through the Cu film and reach out to the underlying a-C layer for graphene crystallization.

*Step IV-* H_2_ gas in the meanwhile reacts with the a-C to form carbon radicals in the presence of Cu catalyst and moves out through the grain boundaries and crystallizes into Graphene. The Graphene formation and etching are simultaneous in the presence of the H_2_ gas based on discussed reactions. For a given annealing time and annealing temperature, the supply of carbon radical depends on the a-C layer thickness and different stress levels, and hence different quality of Graphene is obtained for different samples.

## Conclusion

Various experiments are successfully conducted in this work in order to explore the growth mechanisms of Graphene synthesis using a-C as the carbon source and Cu as the catalyst. Diffusion of H_2_ gas through copper grain boundaries to interact with a-C underneath and thermo-mechanical stress in copper and a-C films are proposed to be the mechanisms for the Graphene synthesis. Therefore, even though samples S1.1 and S1.2 are similar in configuration, their dependence of the graphene growth rate on the annealing time and temperature are different due to their difference in thickness of a-C, which result in different film stresses in the two samples. The proposed growth mechanisms are found to valid for in the temperature range of 1020 to 820 °C. With this mechanism, the infeasibility of Graphene formation on copper film as reported by previous works can also be explained.

## Additional Information

**How to cite this article**: Narula, U. *et al*. Growth Mechanism for Low Temperature PVD Graphene Synthesis on Copper Using Amorphous Carbon. *Sci. Rep.* **7**, 44112; doi: 10.1038/srep44112 (2017).

**Publisher's note:** Springer Nature remains neutral with regard to jurisdictional claims in published maps and institutional affiliations.

## Supplementary Material

Supplementary Information

## Figures and Tables

**Table 1:** Raman Characteristics for annealed samples at 1020 °C for 50 mins^22^.

Sample	I_D_ Peak [cm^−1^]	I_G_ Peak [cm^−1^]	I_2D_ Peak [cm^−1^]	I_D_/I_G_	I_2D_/I_G_	2D Peak FWHM [cm^−1^]
S1 (60 nm a-C)	1362	1584	2726	0.496	0.29	48
S1.1 (36 nm a-C)	1355	1589	2711	0.464	0.89	56
S1.2 (12 nm a-C)	1367	1584	2730	0.117	0.42	52
