# Supplementary material for: Growth Mechanism for Low Temperature PVD Graphene Synthesis on Copper Using Amorphous Carbon
Source: Sci Rep. 2017 Mar 9;7:44112. doi: 10.1038/srep44112 (PMC5343459; doi:10.1038/srep44112)
Supplement: Supplementary Information [file srep44112-s1.pdf]

Supporting Information

# Growth Mechanism for Low Temperature PVD Graphene Synthesis on Copper Using Amorphous Carbon

Udit Narula[1,2], Cher Ming Tan[1,2,3,4,5,*], Chao Sung Lai[2,6,7*]

## 1. Sample Preparation

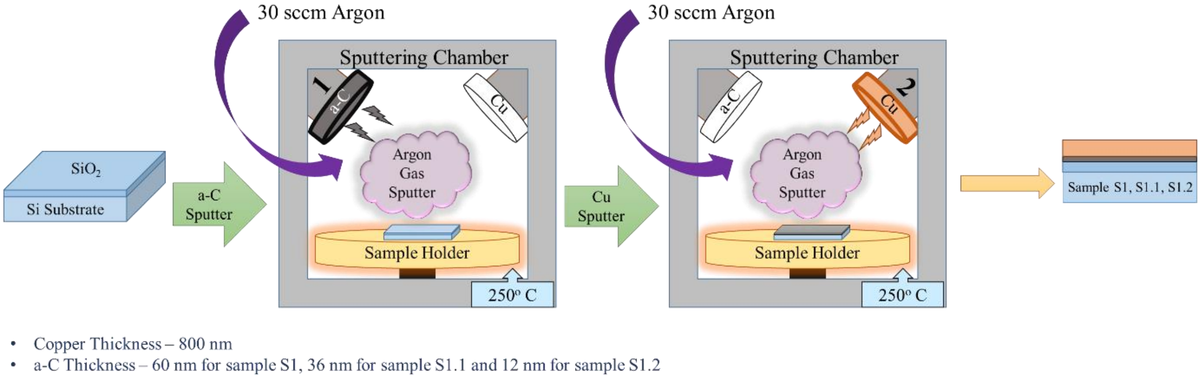


**Supplementary Figure S1**. Sample preparation using PVD method

## 2. Copper Crystal Orientation on Test Structure

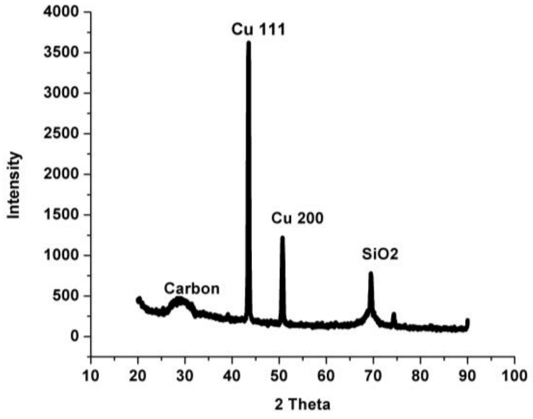


**Supplementary Figure S2**. XRD analysis on deposited Cu surface shows presence of a high intensity <111> Cu peak in XRD analysis result. <111> crystal orientation of Cu is considered to be favorable for high quality single crystal Graphene synthesis[1] as there is minimum lattice mismatch between <111> Cu and Graphene.[2]

### 3. Total Deformation Variation for different annealing temperatures

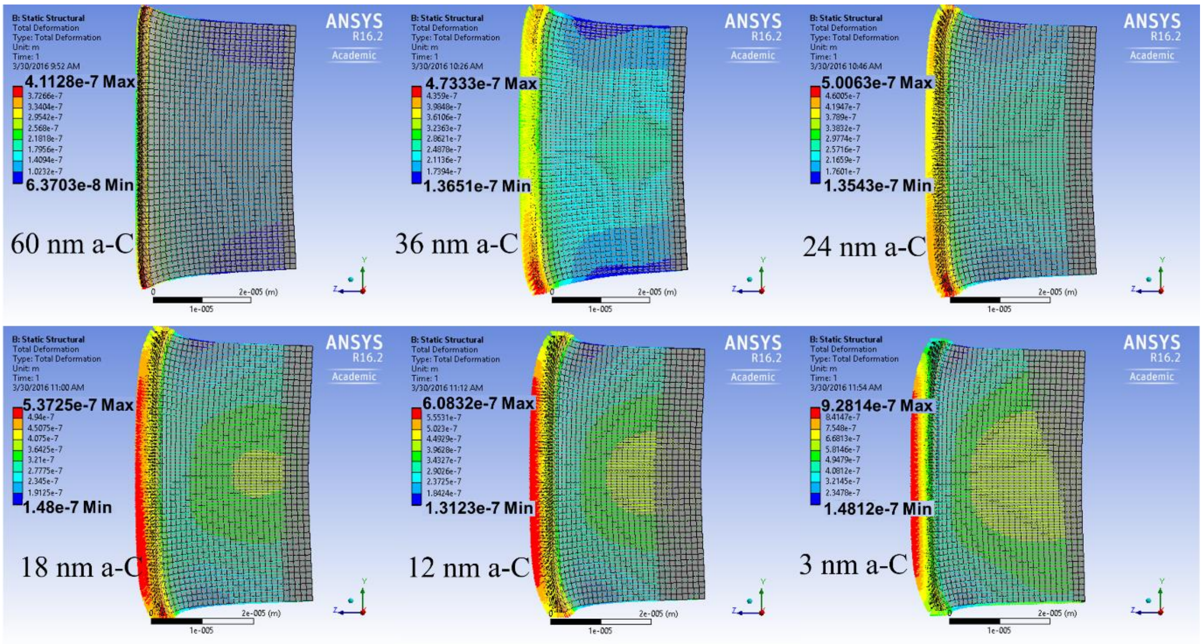


**Supplementary Figure S3**. ANSYS simulation results for annealing temperature value of 1020 $^{o}$C showing total deformation in the samples which migrates from the corners to the center with the decrease in a-C layer thickness.

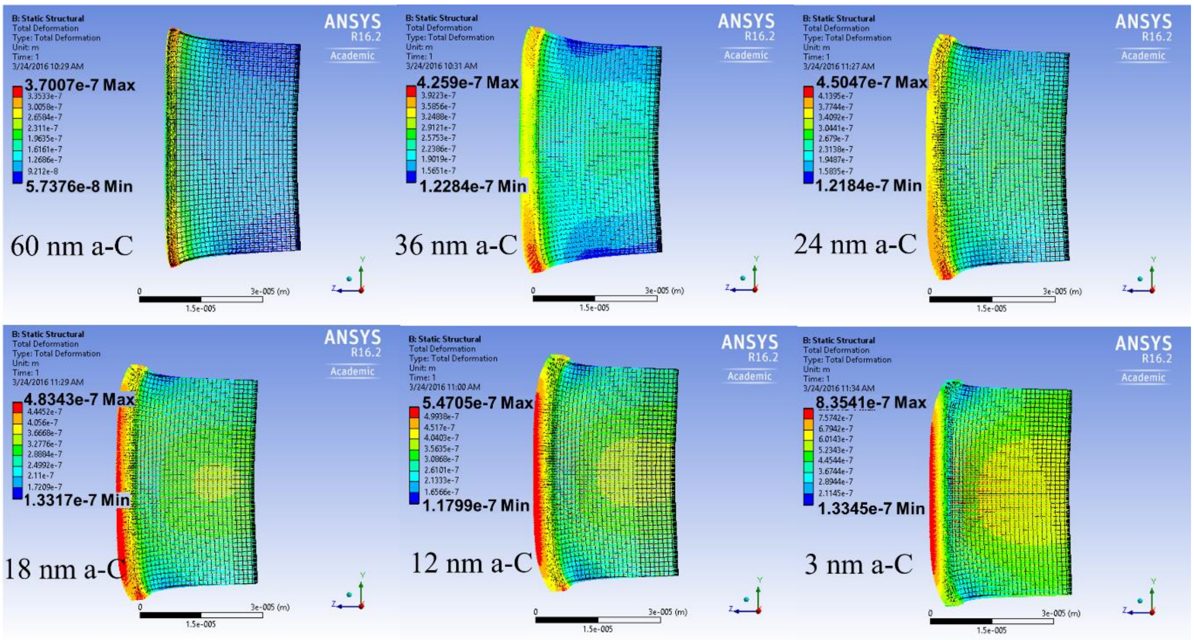


**Supplementary Figure S4**. ANSYS simulation results for annealing temperature value of 920 $^{o}$C showing total deformation in the samples which migrates from the corners to the center with the decrease in a-C layer thickness.

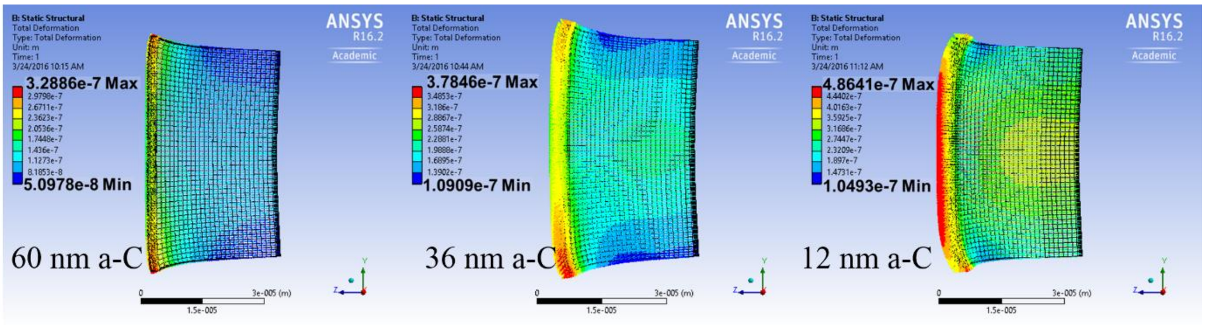


**Supplementary Figure S5**. ANSYS simulation results for annealing temperature value of 820 °C showing total deformation in the samples which migrates from the corners to the center with the decrease in a-C layer thickness.

**Supplementary Table S1**. Thermo-mechanical properties

| **Property** | **$SiO_2$ film** | **a-C film** | **Cu film** | **Graphene** |
|---|---|---|---|---|
| Thermal Coefficient of Expansion [$^{o}C^{-1}$] | *5.0 x $10^{-7}$* [3] | *1.5 x $10^{-6}$* [5] | *1.6 x $10^{-5}$* [7] | *-8 x $10^{-6}$* [9] |
| Young's Modulus [TPa] | *0.070* [4] | *0.759* [6] | *0.115* [8] | *0.96* [10] |
| Poisson's Ratio | *0.1700* [4] | *0.1700* [6] | *0.3430* [8] | *0.1700* [10] |

## Reference

[1] Ishihara, M., Koga, Y., Kim, J., Tsugawa, K. & Hasegawa, M. Direct evidence of advantage of Cu(111) for graphene synthesis by using Raman mapping and electron backscatter diffraction. *Mater. Lett.* **65,** 2864; DOI:10.1016/j.matlet.2011.06.047 (2011).

[2] Terasawa, T. & Saiki, K. Growth of graphene on Cu by plasma enhanced chemical vapor deposition. *Carbon*. **50**, 869; DOI:10.1016/j.carbon.2011.09.047 (2012).

[3] Blech, I. & Cohen, U. Effects of humidity on stress in thin silicon dioxide films. *J. Appl. Phys.* **53**, 4202; DOI:10.1063/1.331244 (1982).

[4] Kim, M. T. Influence of substrates on the elastic reaction of films for the microindentation tests. *Thin Solid Films.* **283**, 12; DOI:10.1016/0040-6090(95)08498-3 (1996).

[5] Marques, F. C. et al. Thermal expansion coefficient of hydrogenated amorphous carbon. *Applied Physics Letters.* **83**, 3099; DOI:10.1063/1.1619557 (2003).

[6] Cho, S. W., Chasiotis, I., Friedman, T. A. & Sullivan, J. Direct measurements of Young's modulus, Poisson's ratio and failure properties of ta-C MEMS. *J. Micromech. Microeng.* **15**, 728; DOI:10.1088/0960-1317/15/4/009 (2005).

[7] White, G. K. Thermal expansion of reference materials: copper, silica and silicon. *J. Phys. D: Appl. Phys.* **6**, 2070; DOI:10.1088/0022-3727/6/17/313 (1973).

[8] Bloomfield, M. O., Bentz, D. N. & Cale, T.S. Stress-induced grain boundary migration in polycrystalline copper. *Journal of Electronic Materials.* **37**, 249; DOI:10.1007/s11664-007-0354-7 (2008).

[9] Yoon, D., Son, Y. W. & Hyeonsik. C. Negative thermal expansion coefficient of graphene measured by Raman spectroscopy. *Nano Lett.* **11**, 3227; DOI:10.1021/nl201488g (2011).

[10] Faccio, R., Denis, P., Pardo, H., Goyenola, C. & Mombrú, A. W. Mechanical properties of graphene nanorribons. *J. Phys: Cond. Matt.*, **21**, 285304; DOI:10.1088/0953-8984/21/28/285304 (2009).
